# Asthma management with breath-triggered inhalers: innovation through design

**DOI:** 10.1186/s40733-020-00057-7

**Published:** 2020-06-06

**Authors:** Mário Morais-Almeida, Helena Pité, João Cardoso, Rui Costa, Carlos Robalo Cordeiro, Eurico Silva, Ana Todo-Bom, Cláudia Vicente, José Agostinho Marques

**Affiliations:** 1 Allergy Center, CUF Descobertas Hospital, Lisbon, Portugal; 2 Allergy Center, CUF Descobertas Hospital and CUF Infante Santo Hospital, Lisbon, Portugal; 3 CEDOC (Chronic Diseases Research Center), NOVA Medical School, Universidade NOVA de Lisboa, Lisbon, Portugal; 4 Pulmonology Department, Centro Hospitalar de Lisboa Central, Lisbon, Portugal; 5 NOVA Medical School, Universidade NOVA de Lisboa, Lisbon, Portugal; 6 Family Medicine, Porto, Portugal; 7 GRESP (Grupo de Estudos de Doenças Respiratórias da APMGF), Lisbon, Portugal; 8 Pulmonology Department, Centro Hospitalar Universitário de Coimbra, Coimbra, Portugal; 9 Faculty of Medicine, University of Coimbra, Coimbra, Portugal; 10 Family Medicine USF João Semana, Aveiro, Ovar, Portugal; 11 GRESP Inhalers and Tecnhical Devices Working Group, Lisbon, Portugal; 12 Immunoallergology Department, Centro Hospitalar Universitário de Coimbra, Coimbra, Portugal; 13 Family Medicine UCSP Soure, Coimbra, Portugal; 14 Pulmonology Department, Centro Hospitalar de São João, Porto, Portugal; 15 Faculty of Medicine, University of Porto, Porto, Portugal

**Keywords:** Asthma, Breath-triggered-inhalers, Control, Inhalers, Innovation, K-haler

## Abstract

**Background:**

Asthma affects the lives of hundred million people around the World. Despite notable progresses in disease management, asthma control remains largely insufficient worldwide, influencing patients’ wellbeing and quality of life. Poor patient handling of inhaling devices has been identified as a major persistent problem that significantly reduces inhaled drugs’ efficacy and is associated with poor adherence to treatment, impairing clinical results such as asthma control and increasing disease-related costs. We herein review key research and development (R&D) innovation in inhaler devices, highlighting major real-world critical errors in the handling and inhalation technique with current devices and considering potential solutions. Furthermore, we discuss current evidence regarding breath-triggered inhalers (BTI).

**Main body:**

The two most common significant problems with inhalers are coordinating actuation and inhalation with pressurized metered-dose inhalers (pMDIs), and the need to inhale forcibly with a dry powder inhaler. BTI R&D plans were designed to overcome these problems. Its newest device k-haler® has several other important features, generating a less forceful aerosol plume than previous pMDIs, with efficient drug delivery and lung deposition, even in patients with low inspiratory flow. The local and systemic bioavailability of fluticasone propionate and formoterol (FP/FORM) administered via k-haler® has been shown to be therapeutically equivalent when administered via the previous FP/FORM pMDI. This device requires very few steps and has been considered easy to use (even at first attempt) and preferred by the patients in a randomized crossover study. In our country, FP/FORM k-haler is available without additional costs compared to FP/FORM pMDI. All devices continue to require education and regular checking of the correct inhalation technique.

**Conclusion:**

BTI R&D can bring advantage over current available inhalers, avoiding the two most common identified critical errors in inhalation technique. K-haler® BTI is currently available, without an increased cost, and approved for adolescents and adults with asthma in whom treatment with inhaled combined therapy with long-acting beta_2_-agonists and corticosteroids is indicated. Its attractive and practical design to facilitate its use has been awarded. K-haler® represents added value through innovation to fulfill actual asthma patient needs, thus with potential relevant impact in asthma management and effective control.

## Background

Asthma affects the lives of several hundred million people around the World, across all age groups, and strongly influences the wellbeing and quality of life of patients [1]. The primary goal of management is to achieve asthma control; this mainly consists of two domains, symptoms control and reduced future risk of adverse outcomes, that are not independent (e.g., well-controlled asthma symptoms significantly reduce future risk of adverse outcomes). Given asthma symptoms’ characteristic variability, asthma management requires regular control assessment, in a close partnership between patients and physicians and other healthcare professionals (HCPs), empowering patients to actively achieve total disease control as a goal. Pharmacological treatments mainly consist of inhaled drugs, allowing efficacy and significantly reducing systemic side effects, being the most efficacious treatments in asthma [1, 2].

Education for health, involving the asthmatic patient and/or caregivers, goes along with other measures to enhance abilities for the inhalation technique and to improve adherence to treatment [1, 2]. In fact, lack of adherence to treatment in asthma occurs in more than half of all medical prescriptions, as in other chronic diseases [2]. However, in asthma and other chronic respiratory diseases, this reduced adherence to treatment allies to an incorrect inhalation technique, both considered related major issues that significantly impair pharmacologic treatments effectiveness [2, 3]. Therefore, despite the efficacy of the available drugs, a high percentage of asthmatics are uncontrolled and have frequent exacerbations.

According to a previous World Health Organization report, interventions that increase adherence to treatment, simplifying it, may have a greater impact than access to new drugs. The adequate knowledge of the barriers to a correct adhesion can allow preventing its occurrence [4].

Nowadays, a broad range of inhalers is available for asthma treatment. Still, many patients with asthma do not use their inhaler correctly [1, 5–9]. Difficulties in using inhalation methods are well-known problems that have been consistently maintained in recent decades. Several errors affect treatment results with the use of both dry powder inhalers (DPIs) and pressurized metered-dose inhalers (pMDIs) [5, 6]. This significantly reduces the amount of drug that is deposited in the airways [10–12], and is clearly associated with poor asthma control [13–18].

Results from the Critikal study analyses of the inhaler technique assessment initiative Helping Asthma in Real-life Patients (iHARP) database have helped to identify the prevalence of critical inhaler errors (those that have a definite detrimental impact on the delivery of drug to the lungs) with different devices in patients with asthma [18]. The most common critical errors included failure to coordinate device actuation and inhalation with pMDIs, and lack of a forceful inhalation with DPIs.

Breath-actuated inhalers (BAIs) represent an evolution in inhalers’ design that may help to improve the management of asthma by reducing the likelihood of these two critical inhaler errors occurring when DPIs and pMDIs are used [19–22].

A novel, ergonomically designed breath-triggered inhaler (BTI), k-haler®, has been developed to effectively improve the delivery of inhaled therapies [23]. K-haler® is not suitable to use with spacers; it produces a slow aerosol plume and is not dependent on actuation-inhalation coordination. Successful use of the k-haler® involves only a few steps and, as an ‘active’ aerosol inhaler, it automatically releases a dose of the drug in a respirable form when the patient inhales, even at a low inspiratory flow. As such, k-haler® represents an added-value to improve asthma control by addressing current patients’ needs and overcoming the most common lasting critical errors referred with pMDIs and DPIs.

## Early history of inhaler devices

Inhalation therapy for respiratory diseases can be traced back thousands of years, but it was later, in the nineteenth century, that most significant developments in inhaler devices took place [24–26]. The first pressurized inhaler was developed in 1858, and further advances in inhalation therapy and inhaler design were achieved in the early twentieth century [25–27]. The precursors of modern inhalers emerged in the 1940s/1950s with the development of the first commercial DPIs and pMDIs [25–28].

## Current DPIs and pMDIs: attributes, advantages and disadvantages

Appropriate inhaler techniques vary according to each device, particularly in the case of DPIs since each has its own specific operation method. DPIs and pMDIs also require different inhalation techniques and specific breathing patterns for optimal drug deposition in the lungs [1, 6, 28]. DPIs rely on the inspiratory force generated by the patient, both to extract the powdered drug from a reservoir or blister and to disaggregate the powder into respirable particles (those with the potential to reach and deposit in the lungs) [18]. By contrast, with pMDIs, the drug (either in a solution or a suspension) is actively expelled from a pressurized canister by a propellant, but coordination of actuation and inhalation is required for its effective delivery to the lungs [18].

A broad range of inhalers is nowadays available for the treatment of asthma, thus the comparative analysis between all existing data is difficult. Although randomized controlled trials (RCTs) have not demonstrated any significant differences in efficacy between device types, most studies were designed to show non-inferiority or equivalence between inhalers [6, 29]. Furthermore, inhaler use in RCTs does not reflect the real-world, where many patients use their inhalers incorrectly [5–9, 18].

## Major issue: many patients do not use their inhaler correctly

The inhaler technique is determinant to achieve asthma symptoms control and a reduced risk of adverse outcomes.

Inhaler handling and inhalation error rates vary considerably across studies, but high rates have been reported for all device types [5, 7, 8, 30]. A study assessing the inhaler technique in 66 individuals with asthma and 90 with Chronic Obstructive Pulmonary Disease (COPD) found that 40% of the patients made at least one potentially serious mistake when using their inhaler device [8]. In an observational study by Melani and colleagues, encompassing 1633 patients who were regularly using inhalers for the treatment of respiratory diseases (asthma, *n* = 864; COPD, *n* = 703; other, *n* = 97), inhaler errors were common with all device types studied, occurring in 12% of patients using pMDIs and 35–44% of those using DPIs (Diskus®, HandiHaler® and Turbuhaler®) [7]. In a systematic review of asthma and COPD studies, the mean percentage of patients who could use their inhaler without mistakes was 63% for pMDIs, 65% for DPIs and 75% for BAIs [29].

However, higher frequencies of incorrect inhaler technique have been reported. Data from a retrospective, observational study of 3654 asthmatics that were enrolled in iHARP showed that, overall, 89% of patients made at least one potentially critical handling error and that 67% made multiple potentially critical errors [18].

## Incorrect inhaler technique is associated with poor asthma control

Incorrect inhaler technique can significantly reduce the amount of drug deposited in the lungs [10–12], and impair drug effectiveness and safety [6, 7, 13–18]. In a prospective observational study by Giraud and colleagues, involving 727 patients with asthma, suboptimal inhaler technique was significantly and independently associated with poor asthma control (Asthma Control Questionnaire [ACQ] score ≥ 1.5; *p* = 0.008, 31]. Data from the study by Melani and colleagues showed an association between poor disease control, assessed by Asthma Control Test™ [ACT] scores, and risk of critical errors in inhalation technique (odds ratio [standard error]; OR [SE] 1.53 [0.14]; *p* < 0.0001). Critical errors were also associated with increased hospital admissions (OR [SE] 1.47 [0.17]; *p* = 0.001), emergency department visits (OR [SE] 1.62 [0.20]; *p* < 0.001), and antibiotics (OR [SE] 1.50 [0.15]; *p* < 0.0001) and oral steroids use (OR [SE] 1.54 [0.16]; *p* < 0.0001, 7]. Different studies have also found that a high frequency of inhaler errors was associated with poorer asthma control, including an increase in the frequency of exacerbations [31, 32]. In a real-world observational study of 3955 patients, 71% used their inhaler incorrectly and, importantly, the number of errors inversely correlated with the level of asthma control (linear regression analysis r = 0.3; *p* = 0.0001, 14].

**Fig. 1:**
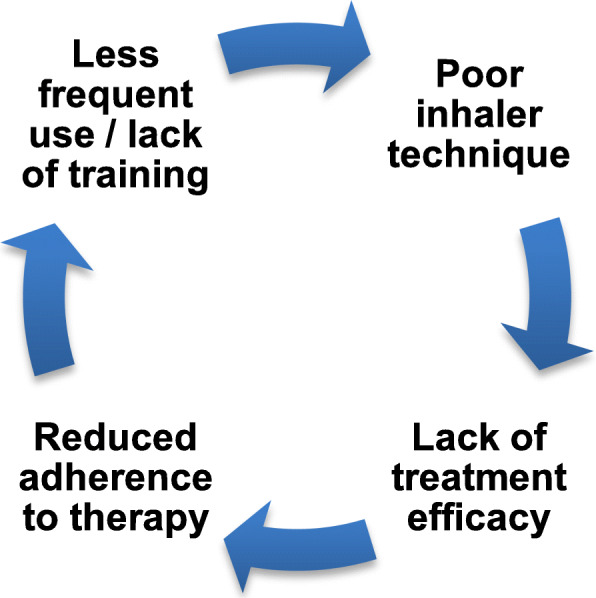
The vicious cycle from inhalation errors to reduced adherence

The effect of handling and inhalation errors on asthma control could result in a vicious cycle, wherein poor inhaler technique leads to frequent symptoms and exacerbations, which in turn can then lead to reduced adherence (Fig. 1). Reduced adherence can also contribute to an inadequate inhaler technique, due to less frequent use and lack of training. HCPs often overestimate the effectiveness of their patients’ inhaler technique [9], therefore potentially compounding the problem by missing opportunities to help patients to improve their inhaler technique.

The development of easy-to-use inhalers that are less prone to inhalation technique errors remains a key clinical need, from mild to severe disease, and including also exacerbations. In addition, a better understanding of which errors have the greatest potential to affect symptoms control would facilitate the development of improved inhalers and/or training programs to overcome these errors.

## Identifying critical errors with DPIs and pMDIs

The nature and frequency of the different errors varies between devices, as would be expected, given that each device requires specific handling and inhalation techniques [31]. Overall, inhalers with fewer steps to operate may be less prone to errors [5]. This highlights the need for easy-to-use inhalers that have only a few operational steps.

Errors can be categorized as either ‘critical’ or ‘non-critical’ [5, 18, 33]. While there are several definitions of critical errors, a review defined critical error as an action or inaction that in itself would have a definite detrimental impact on the drug delivery to the lungs [32]. However, it should be stressed that the identification of critical errors may be largely based on expert opinion and information on the frequency of such errors and their impact on asthma control is limited.

Data from the Critikal analyses of the iHARP database have provided real-world insights into the types and frequency of inhaler errors and their impact on asthma outcomes: A cross-sectional study of 3660 patients with asthma conducted between 2011 and 2014 identified specific inhaler handling errors associated with poor disease control for Diskus®, Turbuhaler® and a pMDI, as well as generic patient factors [18]. This data from the iHARP analysis are consistent with previous studies showing that failing to inhale with sufficient force from the start and poor coordination of actuation and inhalation remain common errors with DPIs and pMDIs, respectively.

### Generic errors

In the Critikal analyses, the most common critical errors were identified as being associated with all inhaler devices (i.e. generic errors) [18]. More than a quarter of patients did not breathe out to empty their lungs as much as possible before inhalation (Diskus®, 32.4%; Turbuhaler®, 26.2%; pMDI, 25.4%). Patients making this error were more likely to have uncontrolled asthma in the previous week (as defined by the Global Initiative for Asthma [GINA] guidelines) than those who did not make the error (OR [95% confidence interval [CI] 1.48 [1.13–1.94], 1.27 [1.06–1.53] and 2.13 [1.57–2.89] for Diskus®, Turbuhaler® and pMDI, respectively).

Another common mistake was not holding breath for at least 3 s following inhalation (pMDI, 33.4%; Diskus®, 24.7%; Turbuhaler®, 22.1%). Insufficient breath-holding was also associated with GINA-defined uncontrolled asthma in the previous week (OR [95%CI] 1.77 [1.34–2.35], 1.96 [1.46–2.63] and 1.53 [1.26–1.85] for pMDI, Diskus® and Turbuhaler®, respectively).

### DPI-specific errors

The Critikal analyses have also shown that an insufficient respiratory effort was one of the critical errors associated with uncontrolled asthma in patients using either the Diskus® or Turbuhaler® DPI. In total, 38.4% of patients using Diskus® and 32.1% of those using Turbuhaler® did not inhale with sufficient inspiratory flow [18].

Multivariate logistic regression analysis demonstrated that patients making this critical error with either Diskus® or Turbuhaler® were more likely to have uncontrolled asthma in the previous week (as defined by GINA) than those who did not make this error (Diskus® adjusted OR [95%CI] 1.62 [1.23–2.14]; Turbuhaler® OR [95% CI] 1.30 [1.09–1.54]; this association remained after taking patient factors into consideration (Diskus® OR [95%CI] 1.56 [1.17–2.07]; Turbuhaler® OR [95%CI] 1.30 [1.08–1.57]).

### pMDI-specific errors

Not coordinating actuation and inhalation appropriately (i.e. actuation occurring before inhalation) was a common error identified in 24.9% of the individuals using a pMDI [18].

Multivariate logistic regression analysis showed that patients who did not coordinate actuation with inhalation correctly were more likely to have uncontrolled asthma in the previous week (as defined by the GINA guidelines) than those who performed this step correctly (adjusted OR [95%CI] 1.43 [1.04–1.98]); this also remained true after taking patient factors into consideration (OR [95%CI] 1.55 [1.11–2.16]).

Although it is ideal for pMDI actuation and inhalation to be coordinated, the requirement for split-second coordination is not as critical as the most important aspect of pMDI inhalation technique: to take a slow (< 60 L/min) and deep inhalation, especially if the actuator has been pressed after the inhalation has begun [34, 35].

## Strengths and limitations of BAIs in asthma management

BAIs are aerosol devices in a closed canister, that cannot be contaminated, like pMDIs, but rather than being activated manually, they automatically release a dose of drug when the patient inhales.

Despite BAIs limitations, namely to be available for a limited range of drugs and the need to shake the suspension before each use, these devices may offer several advantages (Table 1).

**Table 1:** Advantages and disadvantages of breath-triggered inhalers (BTIs)

**Main advantage over pressurized metered dose inhalers (pMDIs)**	
There is no need to coordinate inhalation and actuation, since the device is self-triggered by the patient’s inspiratory flow.	
**Main advantage over dry powder inhalers (DPIs)**	
BTIs release the drug at low inspiratory flow. Therefore, patients do not need to inhale forcibly. Furthermore, drugs’ impact on upper airways is reduced.	
**Further advantages of k-haler BTIs**	
The device is small, light and portable.	
The device allows multiple doses, without any charging.	
It has got a dose counter, so that patients know how many doses are left.	
Few steps are needed to prime and operate the device, making it simple to use.	
Audible “clicks” when priming and closing allow feedback that the device is ready to use or store, respectively.	
The automatic release of a dose when the mouthpiece cap is closed prevents double or multiple doses if a primed dose is not taken.	
The closed canister avoids contents’ contamination.	
The cap is connected to the device, therefore it cannot be lost.	
There is high reproducibility in the amount of drug delivered.	
**Main disadvantages**	
BTIs are available for a limited range of drugs.	
If there is a suspension enclosed, patients need to remember to shake the device before each use.	
BTIs need priming before first use, in case of cold environment or if not used for some time.	

Legend: *BTIs* breath triggered inhalers, *DPI* dry powder inhalers, *pMDI* pressurized metered dose inhalers

BAIs were developed to overcome the most commonly seen critical errors with other inhalers. There is no need to coordinate actuation and inhalation (which is necessary for pMDIs) and, as active devices, BAIs emit a propelled aerosol and patients do not need to inhale forcibly to generate respirable particles (which is required for DPIs), being efficient with low inspiratory flow.

### BAIs are associated with improved inhaler technique and patient preference compared with other inhalers

BAIs are intended to simplify the inhaler technique, leading to improved inhaler use by the patients and less HCP time spent training patients to use the devices correctly [22]. Indeed, several studies have shown that patients find BAIs easier to use and HCPs find it easier to train patients in their correct use in relation to other devices [9, 19, 36].

In an observational, real-life study encompassing 3811 individuals with asthma or COPD, the proportion of patients making at least one critical error with Autohaler® (11%) was significantly lower than with other devices such as a pMDI and the Turbuhaler® DPI (28 and 32%, respectively; *p* < 0.05, 9].

In another study, a greater proportion of individuals with asthma found Easi-Breathe® easier to use correctly than a conventional pMDI (86% vs 14%, respectively; *n* = 104) and nurses found it easier to train patients in the use of the device (99% vs 1%, respectively; *n* = 104) (*p* ≤ 0.001 for both comparisons) [19]. Moreover, 79% of nurses preferred the BAI to pMDIs, with the main reasons being ‘easier for patients to use correctly’ and ‘easier to teach’ (*p* ≤ 0.001). Most patients also expressed a preference for Easi-Breathe® over pMDIs (82% vs 18%; *p* < 0.001); ease of use and confidence in effective dose delivery were stated as the key reasons for their preference. The ease of use and consequent potential for reduced training time with BAIs may be an important advantage, especially given the limited time available in typical primary care clinical consultations. The use of inhalers with no adequate education can lead to more errors, reduced therapeutic compliance and impaired asthma control.

The Sirocco Study, including 6512 patients with asthma, found that 91.4% were able to use the Autohaler® without errors after a median training period of only 4 min, indicating that inhaler training would be feasible in everyday clinical practice [36].

The ease of use of BAIs may offer particular advantages in certain patient groups, such as children, elderly or those with limited manual dexterity [21, 22, 37]. Easy-to-use BAIs that are triggered by a low inspiratory force may offer advantages [21, 22].

The ease of use and patient preference for BAIs may translate into improved treatment compliance with the prescribed therapy [20, 22]. However, this needs to be assessed further with robust RCTs.

### BAIs may help to improve asthma control

Some studies have suggested that use of BAIs may result in improved lung function and asthma outcomes compared with other devices [20–22, 38–41].

A study by Newman and colleagues assessed the efficacy of the Autohaler® in 18 individuals with asthma [38]. Patients who could not coordinate actuation and inhalation with a pMDI (*n* = 8) achieved significant improvements in lung function with salbutamol when they used the Autohaler® compared with the pMDI (*p* < 0.05). Notably, however, lung function was not improved when using the Autohaler® instead of the pMDI in the group of patients with good coordination. Thus, besides demonstrating the potential benefits of BAIs, these data also show that pMDIs are successful airways delivery devices when used correctly.

The clinical efficacy of different inhaler types was assessed in a study in 51 elderly patients with asthma (mean age 77.4 years). Among patients who had a poor inhaler technique, improved clinical responses to salbutamol were observed in most patients who were switched from a pMDI to a BAI (*n* = 10/11), but not in those who were switched from a pMDI to a DPI (*n* = 3/10; *p* = 0.006). Further switching from a DPI to a BAI resulted in improved inhaler technique in five out of seven patients [39].

The SYSTER survey reported significant improvements in asthma control and adherence after 4 weeks of inhaled corticosteroids (ICS) therapy administrated via the Autohaler® in 1510 patients with asthma (who were either initiating therapy or were switched from another device) [20]. After 4 weeks of therapy with the Autohaler®, asthma control had significantly improved compared with baseline (mean [SD] ACQ score: 2.35 [1.05] at baseline vs 1.32 [0.93] at week 4; *p* < 0.0001). Moreover, use of the Autohaler® was associated with significantly increased patient-reported adherence, assessed using the Morisky scale (mean [SD] score 2.11 [1.43] at baseline vs 1.57 [1.53] at week 4; *p* < 0.0001), and satisfaction with the inhaler, assessed using a visual analogue scale (mean [SD] score 51 [19] at baseline vs 75 [14] at week 4; *p* < 0.0001).

The reasons for these differences in outcomes between inhaler types are unclear, but may relate to patient preference, improved adherence and/or better inhaler handling, all of which are reported advantages of BAIs. These data highlight the potential benefits of having easy-to-use devices such as BAIs available in clinical practice.

## The k-haler®: a very unique BTI

The k-haler® is a recent BAI, and is also referred to as a BTI [23]. This term emerged during the development of the k-haler® ‘Instructions for use’, when it became apparent that many patients did not understand the term ‘actuate’ or ‘actuation’, whereas they could easily relate to the concept of the dose release being ‘triggered’ by inhalation. This new term also ensures that the k-haler® is described in a distinct way that clearly separates it from conventional BAIs.

The k-haler® has won an award for its ease of use and patient-focused design, requiring only a few steps to achieve successful drug delivery [23]. This is an ‘active’ device that automatically releases a dose of the drug in a respirable form when a patient inhales, even at a low inspiratory force (Table 1). Thus, this device significantly helps patients to avoid failure to coordinate actuation and inhalation (that is critical when using a pMDI), and not inhaling with sufficient force to produce respirable particles (essential when using a DPI).

Fluticasone propionate/formoterol (FP/FORM) k-haler® contains the same drug components in the same canister as the previous FP/FORM pMDI and it is currently approved for the treatment of adolescents (≥12 years) and adults with asthma [23].

Data from a prospective, cross-sectional survey of patients with asthma and their physicians in the USA suggest that patients’ satisfaction with their inhaler is associated with adherence to therapy, which may lead to improved outcomes in asthma, namely control achievement [42]. With this in mind, k-haler® has several attributes that may be favorable to patients and increase acceptability of the device, from the shape and small size, with the cap connected to the device, to the simplicity of use and the ease of reading the dose.

**Fig. 2:**
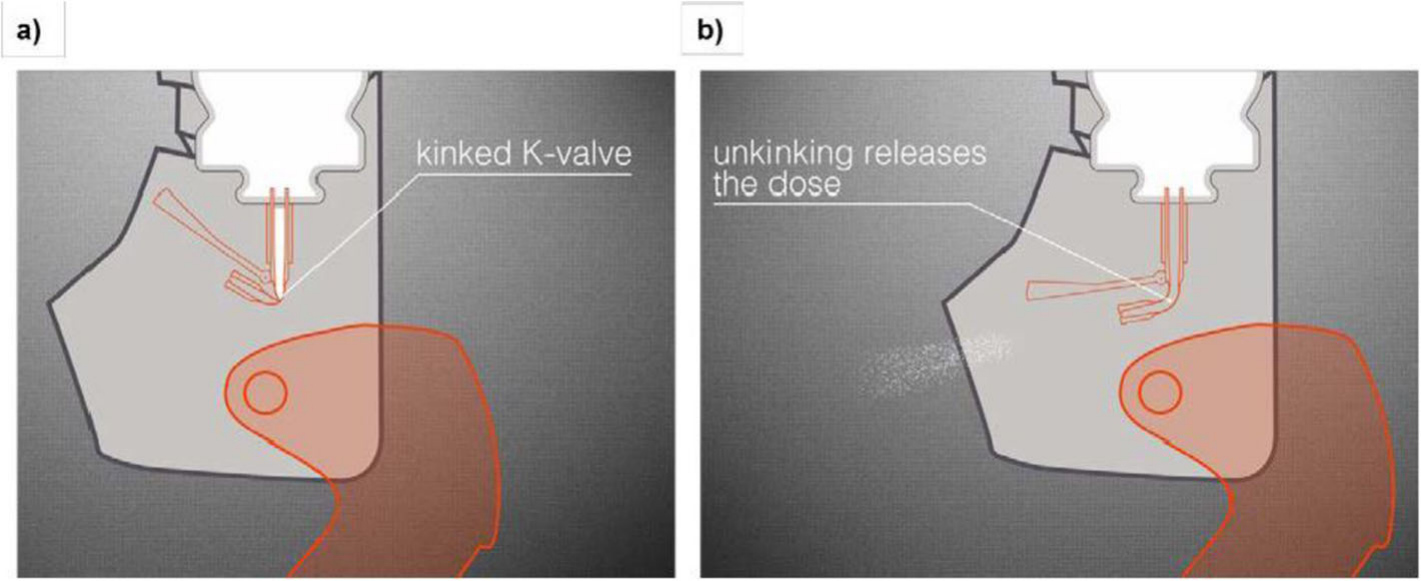
K-haler® K-Valve™ with kinked-hose technology

The innovative K-Valve with kinked-hose technology forms the core of the k-haler® device. When the mouthpiece cap of k-haler® is opened fully, the hose of the K-Valve™ remains kinked (much like a kink in a hose-pipe) and the valve remains closed (Fig. 2a). At the same time, the canister stem is depressed, actuating the device and allowing a single dose of the aerosolized drug to enter the K-Valve™. The device is now primed; because the hose of the K-Valve™ is kinked, the drug is held in this valve.

Catches on a flap inside k-haler® hold the K-Valve™ in the closed position until the patient inhales. Inhalation tilts the flap, which straightens the hose of the K-Valve™ (Fig. 2b), thus opening the valve and releasing the dose through the mouthpiece.

Closing the mouthpiece cap resets the flap and K-Valve™; if the patient does not inhale through the device, the primed dose is released automatically when the mouthpiece cap is closed, acting as a useful alert to the patient that the device has not been triggered.

The patient friendly packaging for FP/FORM k-haler® has been designed with clear, informative diagrams in the patient information leaflet and also on the box for maximum exposure; a Quick Response (QR) code allows to access a demonstrative video. A series of web-based training tools have also been developed to accommodate the various learning styles and preferences of different patients.

K-haler® is easy to use, with a simple procedure that includes few steps [43]: shake; breathe out as slowly and deeply as possible; holding the inhaler upright open the orange mouthpiece cover fully and seal lips around the mouthpiece; breathe in slowly and deeply; hold breath for 5 s at least; remove the inhaler from the mouth and close the cover.

## Documentation of added therapeutic value and place in the treatment of FP/FORM k-haler®

The added therapeutic value, from the public health perspective, is the result of comparisons between two or more therapeutic options in order to identify innovation. Innovation is present when a clearly identified, characterized and validated health need is overcome (partially or totally) by the availability of new technology.

As previously discussed, a significant barrier to an effective treatment of asthma is the inability of the patient to properly use the inhalation device which leads to reduced adherence to treatment and asthma control.

The selection of the comparator medicinal product is intended to document the added therapeutic value of FP/FORM k-haler® in the regular treatment of asthma when it is appropriate to use a drug with this combination (an ICS and a long-acting beta_2_-agonist (LABA)), namely in GINA therapeutic steps three to five [1].

Containing the same drug components in the same canister as FP/FORM pMDI, FP/FORM k-haler® would be expected to have similar characteristics, such as its pharmacokinetic and pharmacodynamic properties, a high fine particle fraction (FPF) and a plume that is less forceful than that of previous pMDIs.

In a study that compared the plume force of FP/FORM 125/5 μg K-haler®, fluticasone propionate/salmeterol xinafoate 125/25 μg (FP/SAL) from the Seretide® Evohaler® pMDI, and 125/25 μg FP/SAL from the Sirdupla® pMDI, the FP/FORM K-haler® plume was 70–87% less forceful than the Seretide® and Sirdupla® plumes, at 60 to 95 mm distances [44]. This can decrease drug impaction at the back of the throat and improve delivery to the lungs.

### K-haler®: ease of use and patient preference

The usability of k-haler® and patient preference for the device compared with their current inhaler was assessed in a randomized, open-label, observational crossover study in 307 patients aged at least 12 years with asthma (*n* = 199 [64.8%]), COPD (*n* = 86 [28.0%]) or asthma and COPD (*n* = 22 [7.2%]) [45]. Individuals were stratified by age (12–17 years, *n* = 66 [21.5%]; 18–65 years, *n* = 166 [54.1%]; > 65 years, *n* = 75 [24.4%]) and forced expiratory volume in 1 s [FEV_1_] (< 60%, *n* = 87 [28.3%]; 60–80%, *n* = 104 [33.9%]; > 80%, *n* = 116 [37.8%]). The primary endpoint was successful device use (defined as the proportion of patients who could perform all eight handling and inhalation steps at their first attempt, using a placebo device). Secondary endpoints included the proportions of patients who were able to generate sufficient inspiratory force to trigger the k-haler® to release a dose, who could successfully perform all ‘critical’ steps and who expressed a preference for k-haler® over their current device. The study showed that k-haler® was easy to use, and that over three quarters of patients successfully triggered the device at their first attempt. Patients also found the k-haler® inhalation technique easy to learn. More than three-quarters of patients were able to use k-haler® successfully at their first attempt. Almost 100% of patients could use the k-haler® correctly within 15 min of initial training. At least 44% of patients found k-haler® easier to use than their current maintenance therapy device (compared with Symbicort® Turbuhaler®, Seretide® Evohaler®, Seretide Accuhaler®/Diskus® and Fostair® pMDI). Approximately 61–71% of patients preferred k-haler® to their current maintenance therapy inhaler [45]. This study demonstrated that k-haler® can be used successfully by patients with asthma, and that many patients prefer k-haler® and find it easier to use than their current device [42, 45].

### FP/FORM k-haler® pulmonary bioavailability

The pulmonary bioavailability of FP/FORM k-haler® versus the FP/FORM pMDI was assessed in a single-dose, randomized, open-label, crossover study in 47 healthy adults [46]. The study was designed to demonstrate that FP/FORM pulmonary bioavailability administered via the k-haler® was no less than when administered via the pMDI without a spacer. A charcoal block was used to prevent gastrointestinal absorption of any swallowed drug. Thus, plasma drug levels reflected uptake via the lungs, and provided a surrogate pharmacokinetic estimate of efficacy compared with that of the pMDI.

The FP/FORM pulmonary bioavailability administered via the k-haler® was similar to the levels achieved when administered via the pMDI without a spacer; hence, the k-haler® would be expected to provide similar efficacy to the pMDI [43–46].

When comparing FP/FORM k-haler® and FP/FORM pMDI, the lower limits of the 94.12% CI for least-squares (LS) geometric mean area under the curve over time (AUCt), area under the curve to infinite time (AUCINF) and maximum serum concentration (Cmax) exceeded the pre-specified lower limit of 80%, indicating similarity between the devices, when appropriate inhalation techniques are ensured [46].

Furthermore, the concentration-time profiles for FP/FORM were similar when administered via the k-haler® and via the pMDI indicating similar regional pulmonary bioavailability patterns. FP/FORM k-haler® was generally well tolerated, with a similar adverse event profile to that of FP/FORM pMDI [43, 46].

### FP/FORM k-haler® total systemic bioavailability

The total systemic bioavailability of FP/FORM when administered via the k-haler® and via the pMDI were assessed in a single-dose, randomized, open label, crossover study in 48 healthy adults [47]. The primary objective of the study was to assess the total systemic exposure (as measured by Cmax, AUCt and AUCINF) of FP/FORM administered via the k-haler® 125/5 μg (two puffs, 250/10 μg total dose) and the pMDI 125/5 μg (two puffs, 250/10 μg total dose), with and without a spacer.

The study demonstrated that the systemic bioavailability of fluticasone propionate administered via the k-haler® was not greater than when administered via the pMDI with a spacer, and that the systemic bioavailability of formoterol administered via the k-haler® was similar to that administered via the pMDI without a spacer [43, 47].

When comparing fluticasone propionate administered via the k-haler® and via the pMDI with a spacer, the upper limits of the 94.12% CI for LS geometric mean AUCt, AUCINF and Cmax were below the pre-specified safety threshold of 125%, indicating equivalent exposure with the two devices [47].

For formoterol administered via the k-haler® and the pMDI without a spacer, the upper limit of the 94.12% CI for AUCt, and AUCINF ratios were below the pre-specified safety threshold of 125%; however, the corresponding value for Cmax was narrowly outside this threshold (125.97%).

Because the exposure of formoterol administered via the k-haler® was greater than that via the pMDI without a spacer, the study was extended to include pharmacodynamics assessments of formoterol-mediated effects, in order to definitely confirm the safety of the formoterol component in FP/FORM k-haler®.

These further analyses demonstrated that the k-haler® was equivalent to the pMDI with a spacer for maximum reduction in serum potassium (ratio [95%CI] 0.97 [0.73–1.28]; primary endpoint). These data confirm that the formoterol-related systemic safety of FP/FORM k-haler® is similar to that of FP/FORM pMDI [43, 47].

### FP/FORM k-haler® scintigraphy study and lung deposition

FP/FORM k-haler® and FP/FORM pMDI are expected to produce similar lung deposition when used correctly. Previous studies using in vitro functional respiratory imaging have suggested that FP/FORM pMDI provides drug lung deposition of up to 44% of the metered dose in patients with asthma (based on modelled data) [48], which is notably greater than that seen with other ICS/LABA combinations using the same methodology [49, 50].

A recent study assessed the in vivo lung deposition of FP/FORM k-haler® using scintigraphy [51]. Following training on how to use the k-haler® correctly, a total of 36 healthy volunteers, patients with asthma (GINA step 2 or higher, FEV_1_ ≥ 60% and ≤ 90% predicted) and patients with COPD (FEV_1_ of ≥30% and ≤ 50% predicted) (12 individuals in each group) inhaled a radiolabeled (99mTc) suspension of FP/FORM via k-haler®.

FP/FORM k-haler® provided high levels of mean (SD) lung deposition of 44.7% (14.2) in patients with asthma, and of 39.0% (13.0) and 26.6% (8.0) in patients with COPD and healthy volunteers, respectively. This demonstrates that the k-haler® is associated with efficient drug delivery in vivo that is similar to the modelled lung deposition observed with FP/FORM pMDI [43, 51].

Importantly, the study showed that lung deposition with FP/FORM k-haler® is not affected by airflow limitation; the increased lung deposition observed in patients with asthma or COPD compared with healthy volunteers is probably due to these patients having real-life experience in using inhalers [51].

## Analysis of economic value

Adult asthma represents a very significant load of total health expenditure [52]. A considerable proportion is related to the treatment of asthma in uncontrolled patients, since in these cases direct costs are twice as expensive as the cost of treatment in controlled patients [53]. Therefore, therapeutic alternatives with a lower price and that reduce the direct costs to the health systems (costs related to exacerbations, hospitalizations, time spent by HCP) have an economic advantage [54].

In our country, the market price of FP/FORM K-haler®, the first BTI class inhaler containing an ICS/LABA association for the treatment of asthma, allows an economic advantage. This results from a lower price compared to the available direct alternative, as well as from other significant economic advantages that can result from the reduction of risks related to handling errors with the use of inhalers and the reduction of the time that the HCP needs to spend to educate / train the patient to use the inhaler correctly [33, 55].

Using the data from the Critikal study [18], Forsters et al. explored the cost-effectiveness of an error-targeted intervention related with DPI, using a probabilistic Markov cost-utility model, with simulated patients transitioning between controlled and uncontrolled health states over 1 year. The analysis explored complete/partial eradication of the error when the intervention was priced to match comparators, as well as impact of indirect costs based on lost/reduced productivity. This study demonstrated the economic and societal costs of ‘insufficient inspiratory effort’ and potential economic benefits of introducing an effective intervention to reduce/eradicate this error related with DPIs [56]. Thus, this analysis reinforced that inefficient use of inhalers have significant economic consequences.

## Conclusions

The development of easy-to-use inhalers that are less prone to inhalation technique errors remains a key clinical need in respiratory diseases, such as asthma or COPD. BTI R&D brings innovation with advantage over current available inhalers, avoiding the two most common identified critical errors in inhalation technique.

The latest BTI FP/FORM k-haler® contains the same drug components in the same canister as FP/FORM pMDI. It shares many of the same characteristics as the pMDI, such as a high FPF and favorable plume characteristics. Pharmacokinetic studies have shown that FP/FORM k-haler® provides a drug delivery similar to FP/FORM pMDI (with and without a spacer), whether assessed by pulmonary or total systemic exposure, demonstrating that the two devices are therapeutically equivalent. Moreover, many patients find k-haler® easier to use than their current device and express a preference for this inhaler. These data are important because patient inhaler satisfaction may be correlated with treatment adherence and improved outcomes in asthma.

High levels of lung deposition have been demonstrated with FP/FORM k-haler® use, highlighting its efficient drug delivery. This inhaler has an attractive design, which has already been awarded. It is simple to use from the first attempt and it is accompanied by appealing materials to support its correct use, accessible either in the handling of an on-site demonstration kit of the prescription (k-trainer), or in the package leaflet, as well as those that are available interactively on the web.

This compact inhaler of reduced size, easy to handle and use, is also suitable for asthmatics with orthopaedic degenerative diseases of the hands. A color-coded front dose meter facilitates asthma management and alerts to the need for inhaler renewal.

In daily practice, on “the real world”, educational measures remain essential, always repeating the cycle: to know, to show, to educate (shake, open, inhale, close), to check and correct any handling or inhalation errors in every medical appointment, and not only in the first clinical consultation. Not only medical doctors but also nurses, pharmacists, lung technicians, the whole healthcare team is called into action to guarantee a continuous successful treatment. If correctly used, inhalers are excellent, safe and effective in controlling asthma. Patients’ preferences should always be respected.

FP/FORM k-haler® simplifying procedures with cost reduction, is already available as a relevant contribution to increase treatment adherence and to facilitate asthma control in adolescents and adults, where treatment with an ICS/LABA association is indicated.

## Data Availability

Not applicable.

## Abbreviations

ACQ: Asthma control questionnaire
ACT: Asthma control test
AUCINF: Area under the curve to infinite time
AUCt: Area under the curve over time
BAI: Breath-actuated inhaler
BTI: Breath-triggered inhaler
CI: Confidence interval
Cmax: maximum serum concentration
CPOD: Chronic pulmonary obstructive disease
DPI: Dry powder inhaler
FEV_1_: Forced expiratory volume in one second
FPF: Fine particle fraction
FP/FORM: Fluticasone formoterol
FP/SAL: Fluticasone salmeterol
GINA: Global Initiative for Asthma
HCP: Healthcare professionals
ICS: Inhaled corticosteroid
iHARP: initiative Helping Asthma in Real-life Patients
LABA: Long acting beta_2_-agonist
OR: Odds ratio
pMDI: pressurized metered-dose inhaler
QR: Quick response
RCT: Randomized controlled trial
R&D: Research and development
SD: Standard deviation
SE: Standard error
